# Spatiotemporal analysis of the effects of exercise on the hemodynamics of the aorta in hypertensive rats using fluid-structure interaction simulation

**DOI:** 10.2478/jtim-2023-0140

**Published:** 2024-03-21

**Authors:** Yueshen Wang, Haiyi Yu, Quanyou Shi, Ming Xu, Wei Gao

**Affiliations:** Department of Cardiology and Institute of Vascular Medicine, Peking University Third Hospital; State Key Laboratory of Vascular Homeostasis and Remodeling, Peking University; NHC Key Laboratory of Cardiovascular Molecular Biology and Regulatory Peptides, Peking University; Beijing Key Laboratory of Cardiovascular Receptors Research, Beijing 100191, China

**Keywords:** exercise, hypertension, hemodynamics, fluid structure interaction model, shear stress

## Abstract

**Background and Objective:**

Hemodynamic changes that lead to increased blood pressure represent the main drivers of organ damage in hypertension. Prolonged increases to blood pressure can lead to vascular remodeling, which also affects vascular hemodynamics during the pathogenesis of hypertension. Exercise is beneficial for relieving hypertension, however the mechanistic link between exercise training and how it influences hemodynamics in the context of hypertension is not well understood.

**Methods:**

n exercise model was developed using spontaneously hypertensive rats (SHR) subject to a 12-week treadmill training regime. The heart rates and blood pressures of rats were measured using the tail cuff method, while micro-computed tomography (CT) scanning was used to develop three-dimensional structures of rat aorta, and ultrasound was used to detect rat aortic blood flow and changes to vessel wall structures. Computational fluid dynamics (CFD) and fluid-structure interaction (FSI) models were used to simulate and measure hemodynamic parameters of the rat aortic vessels. In parallel, Masson staining was performed on fixed samples of blood vessels to investigate collagen volume fraction. Hypertensive rats in the sedentary and long-term exercise training groups were subjected to a single bout exercise training, and their aortic hemodynamic parameters were analyzed before, 5 min, 24 h, and 72 h after the single bout exercise.

**Results:**

Of the two models, in comparison to actual ultrasonic measurement values recorded, we found that numerical simulation results from the FSI model could more accurately model blood flow in the ascending aorta of hypertensive rats, compared to the CFD model. Moreover, longterm exercise training improved local hemodynamic parameters of blood vessels, and led to improvements in adverse hemodynamic features documented, including time-averaged wall shear stress (TAWSS), oscillatory shear index (OSI), and relative residence time (RRT). Longterm exercise training of SHR also improved local vascular collagen deposition in the aorta, while improvements in vascular remodeling were also correlated with favorable hemodynamic parameters. Compared with sedentary SHR, signals for low TAWSS regions of the aortic arch in SHR on the long-term exercise regime shifted to the position of the ascending aorta after a single bout of exercise.

**Conclusions:**

This study demonstrates that FSI is informative to study the spatiotemporal effects of long-term exercise training on hemodynamic changes within the aortas of hypertensive rats, and that long-term exercise is beneficial through its effects to modulate vascular hemodynamics in hypertension.

## Introduction

Hypertension is a leading cause of cardiovascular disease (CVD) morbidity and mortality worldwide, affecting more than 1 billion adults.^[1,2]^ Despite its prevalence, managing hypertension remains a challenge due to a lack of understanding of the underlying causes and pathological mechanisms. Growing evidence suggests that hypertension leads to changes in hemodynamics, particularly at vascular curvatures and branches,^[3]^ which contribute to local vascular remodeling. This, in turn, drives a pathological process that leads to atherosclerotic plaque rupture.^[4,5]^ However, deciphering the mechanisms underlying the hemodynamic environmental changes that contribute to arterial damage remains challenging. At present, computational fluid dynamics (CFD) is commonly used to simulate and calculate the hemodynamic features of the aorta. This method is based upon the assumption that the aortic wall is a rigid wall, and this assumption also represents the main limitation of CFD that precludes its ability to accurately reflect the interaction between blood flow and vascular remodeling. Related to this issue, the application of fluid-structure interaction (FSI) as a method to investigate the hemodynamic changes within the human aorta has recently been reported, and the results showed that FSI modelling takes into account vascular wall elasticity, such that wall shear stress (WSS) exhibited a very different pattern.^[6]^ Despite this progress, the relationship between vascular remodeling and local hemodynamic parameters under conditions of elevated blood pressure remains poorly characterized.

Exercise training plays a crucial role in protecting against cardiovascular diseases, as well as delaying the hallmarks of vascular remodeling.^[7]^ However, the underlying mechanism remains for its effects on vascular remodeling remain to be defined. In a previous study, we utilized micro-computed tomography (CT) imaging and CFD simulation and found that aortic remodeling was evident in hypertensive rats at a level that met the threshold standard for effective anti-hypertensive treatment, and that hemodynamical parameters at the vulnerable regions of the aorta in hypertensive rats were closely related to vascular remodeling.^[8]^ Nevertheless, how to reverse the hemodynamical changes at the vulnerable regions of blood vessels in the context of hypertension, and whether exercise can change the hemodynamical environment of the vulnerable regions of blood vessels during hypertension have not been reported. Therefore, by analyzing the spatiotemporal changes of local hemodynamical parameters in vulnerable areas of the aorta after exercise, we can provide insights into how exercise training could improve hypertension and cardiovascular health.

In this study, we applied FSI simulation to evaluate the effects of exercise on hemodynamical parameters, including time-averaged wall shear stress (TAWSS), oscillatory shear index (OSI), and relative residence time (RRT) of the vulnerable areas of the aorta in hypertensive rats. Also, we explored the relationship of each of these parameters with vascular remodeling. By analyzing the changes in the distribution of low shear stress areas, the spatial impact of exercise training on the distribution of vascular shear stress in the vulnerable areas was demonstrated. By comparing the effects of a single bout of exercise on the hemodynamical parameters of the aorta of hypertensive rats in the sedentary and long-term exercise training groups, we demonstrated the time effect of exercise on the local hemodynamical parameters of the hypertensive aorta. Finally, we demonstrated the protective effect of exercise training on the vascular hemodynamical environment during hypertension.

## Methods

### Animals and exercise protocol

**Figure 1:**
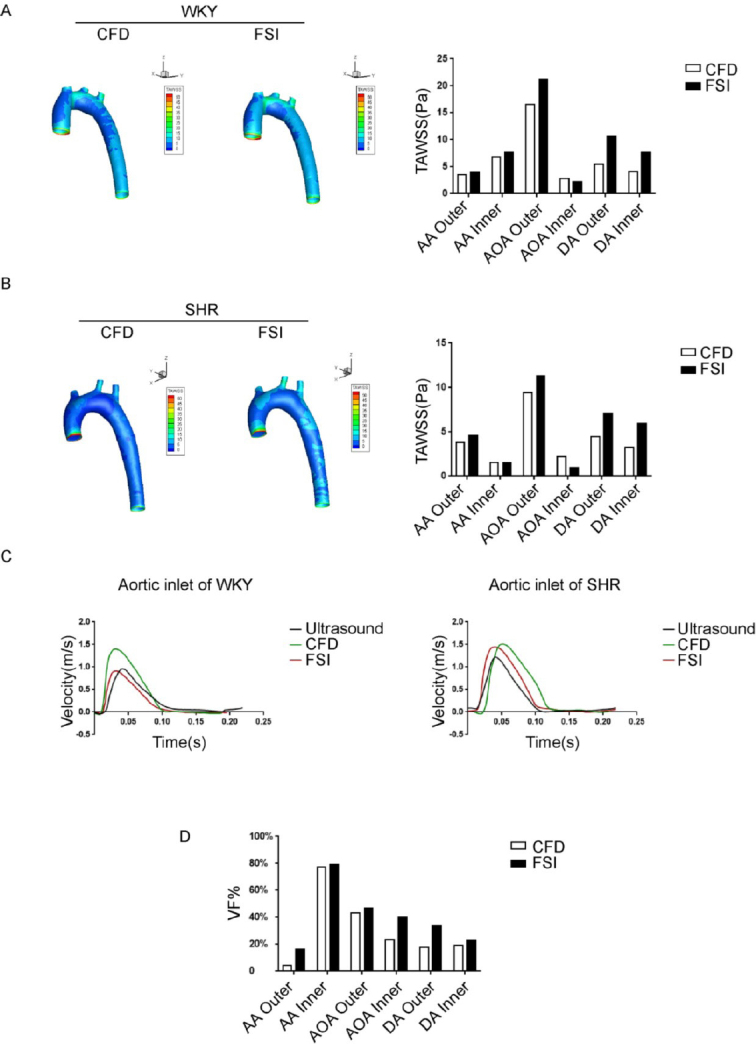
Evaluation of computational fluid dynamics (CFD) and fluid-structure interaction (FSI) methods in simulating aortic hemodynamic properties. (A) Time-averaged wall shear stress (TAWSS) distribution map of the aorta in Wistar-Kyoto (WKY) rats calculated by the FSI method and the CFD method. (B) TAWSS distribution map of the aorta in spontaneously hypertensive rats (SHR) calculated by the FSI method and CFD method. (C) Velocity profiles obtained from ultrasound, CFD, and FSI at the inlet of the aorta. (D) The aortic TAWSS variation fraction (VF %) of SHR relative to WKY rats was calculated using FSI and CFD methods, VF% = (TAWSS_SHR_-TAWSS_WKY_) × 100% / TAWSS_WKY_. AA, ascending Aorta; AOA, aortic arch; DA, descending aorta; WKY, Wistar-Kyoto; SHR, spontaneously hypertensive rats; Outer, the outer wall of aorta; Inner, the inner wall of aorta; TAWSS_SHR_, time average wall shear stress of SHR; TAWSS_WKY_, time average wall shear stress of WKY.

Male spontaneously hypertensive rats (SHR) and Wistar-Kyoto rats (WKY) were purchased from Beijing Vital River Laboratory Animal Technology Corporation (Beijing, China). Three-month-old SHR were randomly divided into the long-term exercise training group (*n* = 12) and the sedentary group (*n* = 12) (Figure 1A). After 3 months of exercise training, the changes in aortic fluid dynamics parameters of hypertensive rats after a single bout exercise were evaluated at 4 time points. Therefore, each group of rats was divided into 4 subgroups, including pre-exercise, 5 min after exercise, 24 h after exercise, and 72 h after exercise groups, with 3 rats in each group.

The exercise protocol for the long-term training group is as follows: formal exercise training begins after a 2-week adaptation period. During the adaptation period, the exercise speed gradually increased from 10 m/min to 18 m/ min, and the exercise time increased from 15 min per day to 60 min per day. During the formal exercise training, rats were trained to run at a speed corresponding to 55%–60% VO_2_ max (18 m/min)^[9]^ for 1 h each day, 5 days a week (Monday to Friday), for a period of 3 months.

To evaluate the difference in protective effects of a single bout of exercise between the long-term exercise training group and the sedentary group, we administered a single bout of exercise training (18 m/min, running for 1 h) to the two groups of rats. FSI simulations were performed on local aortic hemodynamic indicators at 5 min, 24 h, and 72 h after the end of the single bout of exercise.

### Ultrasound imaging

To perform ultrasound imaging on a rat, the rat’s chest is first shaved using hair removal cream (Nair, Mississauga, Canada). The rat is then anesthetized with 2% isoflurane (1 L/min) in a closed anesthesia chamber until the paw-pinch reflex disappeared. The rat is then quickly placed in a supine position on a heating pad and a nose cone is placed over its nose to maintain anesthesia with 2% isoflurane. Ultrasound imaging was performed using a Vevo 2100 ultrasound system (Fujifilm Visualsonics, Toronto, ON, Canada). Three consecutive cardiac cycles were analyzed to measure relevant parameters. The ultrasound probe was then moved to the left edge of the rat’s sternum and the fine-tuning screw was used to adjust the signal so as to obtain the long axis view of the aorta, with the sampling line parallel to the long axis. The blood flow velocity wave form of the ascending aorta (AA) was obtained using Doppler mode. The ultrasound probe is then rotated 90° to display the aortic arch (AOA) at the level of the sternal notch, with the sampling line parallel to the direction of blood flow. The blood flow velocity wave form of the proximal descending aorta was obtained using Doppler mode, which was used as a boundary condition for CFD calculations.

Ultrasound was also used to detect changes of vessel wall structures. The ultrasound probe was positioned perpendicularly at the left edge of the sternum of rats, and then translated to the subclavian region below the rat’s clavicle. The probe angle was adjusted horizontally to obtain a clear two-dimensional ultrasound view of the aortic arch. The sampling line was placed between the right brachiocephalic artery and the opening of the left carotid artery, capturing an M-mode ultrasound image. Image analysis was conducted at the ex vivo workstation, measuring the inner diameter of the vessel and the thickness of the intima-media layer. Three consecutive measurements were taken and averaged for analysis.

### Geometrical reconstruction

Rats were anesthetized with tribromoethanol (0.25 mg·g^-1^ intraperitoneal injection), and the common carotid artery was isolated and exposed. The chest was opened and 100 mL of heparin saline (100 U/mL) was immediately injected, and the common carotid artery and abdominal aorta were cut at the same time. After the effluent liquid became transparent, 30 mL of 10% paraformaldehyde was injected. Then 10 mL of casting compound (MV122; Flow Tech, Carver, Mass, USA) was slowly injected. After the castables solidified, aortic images were collected through micro-CT and inputted into the Mimics Materialize software^[10]^ (Mimics Materialize, Ann Arbor, MI, USA) to obtain a three-dimensional reconstructed image. We used Solidwork (Solidworks Inc., USA) to preliminarily process the original CT data of the hypertensive rats. Non-smooth areas were deleted, the vascular inlet and outlet planes were segmented, and the data was saved as an stereolithography (STL) model, imported into SimVascular (open source, URL: http://simvascular.github.io/), and then the model was smoothed. We then performed 10 decimation and constrained smoothing operations to smooth out the model and obtained a solid model that can be used for grid drawing.

### Boundary conditions

The fluid boundary conditions of the AA were assumed as velocity inlet, and velocity outlets were set at AOA branches in the fluid domain. The boundary conditions of the descending aorta were deemed a velocity outlet based on previous experiments, by using high-frequency ultrasound to obtain the boundary conditions in the AOA of rats.

The fluid-solid interface was applied as a boundary condition at the inner surface of the solid domain in the FSI simulations: pressure distribution derived from the solution of the fluid domain was used as external loading on the arterial wall. A zero-displacement boundary condition was set at each inlet and outlet.

### Material parameters and related indicators

Blood was considered as a continuous, homogeneous, and incompressible Newtonian fluid.^[11]^ The blood flow was described by incompressible Navier–Stokes equations and continuity equations. In this study, density and viscosity were considered as 1060 kg/m^3^ and 0.0035 Pa·s respectively.^[12]^ In the structure domain, the vascular wall was set to be linear and isotropic, with Poisson’s ratio of 0.49, Young’s modulus of 7.5 × 10^5^ Pa, and density of 1150 kg/m^3^.^[13]^

### Wall shear stress

Wall shear stress (WSS, *τ⃗_w_*) is the dynamic friction force between a viscous fluid flow and a solid wall, expressed in Pa. TAWSS is the average value of WSS after integration within a cardiac cycle.

$$\begin{array}{c} TAWSS=\frac{1}{T\int_{0}^{T}|{\bar{\tau }}_{w}|dt} \end{array}$$

T is the period of the cardiac cycle time.

### Oscillatory shear index

OSI is used to describe the degree of change in WSS or blood flow direction, and is a dimensionless value between 0 and 0.5.^[14]^ The expression is:

$$\begin{array}{c} OSI=0.5(1-\frac{|\int_{0}^{T}{\bar{\tau }}_{wdt}}{\int_{0}^{T}|{\bar{\tau }}_{w}|dt}) \end{array}$$

### Relative residence time

RRT can be used to evaluate blood flow residence time and local low and oscillatory shear stress.^[15]^ The expression is:

$$\begin{array}{c} RRT=\frac{1}{\text{TAWSS}(1-2OSI)} \end{array}$$

### Computation procedures

FSI simulation: The blood vessel wall data was imported into Meshmixer software (Autodesk Inc., San Rafael, CA, USA), the blood vessel wall was set to 0.2 mm. A solid model was obtained after performing an extruding operation, and then re-import into integrated computer engineering and manufacturing (ICEM) (ANSYS, Inc., Canonsburg, PA, USA) to generate fluid and structure domains. The boundary layer grid used for the next calculation was made in the fluid domain. Simulations were run in ANSYS Workbench (ANSYS, Inc., Canonsburg, PA, USA). The smooth no-slip boundary condition was applied to all walls, and the dynamic mesh was set to the coupling system. Concerning the fluid dynamics analysis, the time step was 200, limited to 20 iterations per time step. Maximum convergence residuals were set for both solvers as 10^–4^.

CFD simulation: On the basis of the same animal vascular casting, the CT image was imported into the Mimics Materialize modeling software (Mimics Materialize, Ann Arbor, MI, USA) to obtain the real geometric model of the animal blood vessel. And on this basis, the ICEM software was used to draw the grid and the calculation grid for CFD simulation.

### Blood pressure measurements and vascular structure analysis

The blood pressure of rats was measured using the tail-cuff method (BP-98A, Softron, Tokyo, Japan). All animals were fitted to the device 3 days in advance before recording. At the time of recording, the rats were in a warm, restrained, and conscious state, with at least 5 consecutive measurements taken, and the average value was used to determine the arterial blood pressure.

The aorta of rats was sampled for quantitative analysis of vascular structure. The aorta was removed and fixed with 4% paraformaldehyde, and dehydrated in 30% sucrose. In addition, the ascending aorta and aortic arch were separated and inserted into the optimal cutting temperature compound in inverted cutted eppendorf (EP) tubes. The marker could indicate the exact location of the outer and inner arterial walls. Serial sections (8 μm thick) were stained with the Masson staining. Image Pro Analyzer software (Media Cybernetics, Rockville, MD, USA) was used for image analysis to quantify aortic cross-sectional area, diameter, vascular wall thickness, and collagen volume fraction.

### Statistical analysis

Statistical analyses were performed using SPSS 26.0 (IBM Corp., Armonk, NY, USA). For data following a normal distribution, such as vascular diameter, intimamedia thickness, vascular structural data obtained from histological staining, and baseline blood pressure data, we employed the Student’ s unpaired *t*-test to analyze the statistical significance between the two groups. The results are presented as means ± standard deviations (SD). In the case of abnormal distribution or when the number of replicates in each group is 3, the Mann-Whitney *U* test was selected to analyze the statistical significance of two groups, and data were presented as median (minimum-maximum). Pearson correlation analysis was used to investigate the correlation between hemodynamic indicators and collagen volume fraction. Two-sided *P* < 0.05 was considered statistically significant.

## Results

### Evaluation of FSI and CFD simulations in a mathematical simulation of aortic hemodynamics parameters in WKY And SHR

Simulation of rat aortic hemodynamics was conducted with both CFD and FSI simulations, with the goal to provide a detailed comparison between the two methods, using actual ultrasound measurements recorded from mouse aortas as a guide. The main differences observed between the simulations developed from FSI and CFD approaches were detected within the AOA region (Figure 1A, 1B). Under pulsatile flow conditions, TAWSS values calculated by FSI simulation was lower at the inner wall of the AOA region, compared to CFD simulation (FSI: 16.61 Pa *vs*. CFD: 21.23 Pa). When comparing simulation results from CFD or FSI to ultrasound measurements, signals for FSI-simulated flow velocity were closer in approximation to ultrasound measurements, compared to the more-distantly related CFD simulation data (Figure 1C). Specifically, in the WKY group, the peak velocity measured by ultrasound was 0.96 m/s, whiles the simulated peak velocities were 0.91 m/s for FSI and 1.40 m/s for CFD. In the SHR group, peak velocity for ultrasound measurements were 1.22 m/s, while simulated peak velocities were 1.45 m/s for FSI and 1.51 m/s for CFD. In addition, FSI aligned with ultrasound regarding the result of peak time, with a result of 0.040 s, while CFD simulated a delayed peak time of 0.051 s.

More detailed comparison of the CFD and FSI results are presented, as follows. The branch vessel and the AA region has lower WSS when simulated using the CFD model, and the results were about 10% lower in values compared with data from the FSI simulation. In addition, the WSS of the outer wall of the AOA, which is distributed in a higher shear stress area, is 20%–25% higher under the FSI simulation model compared to the CFD simulation model, and the area of the high shear stress area was relatively larger when modelled using FSI simulation.

In addition, we calculated values for the TAWSS variation fraction (VF) in the aorta between the SHR and WKY rats (VF % =[TAWSS_SHR_ - TAWSS_WKY_] × 100% / TAWSS_WKY_). Comparing the differences between the FSI and CFD simulations in evaluating TAWSS changes caused by hypertension, at positions such as the AA, AOA, and outer side of the descending aorta, the changes in TAWSS caused by hypertension calculated by the FSI method were more significant than when calculated using the CFD simulation method, indicating that modelling using the FSI simulation method is more sensitive than the CFD simulation method for hypertension, especially for measuring the outer side of the AA (CFD *vs*. FSI: 4.4% *vs*. 16.3%) as well as the inner side of the AOA (CFD *vs*. FSI: 23.2% *vs*. 40.5%) (Figure 1D).

### Evaluating the effect of exercise training on local TAWSS in the aorta of hypertensive rats using FSI

In the preliminary experiment, we provided moderate intensity exercise training to hypertensive rats on a treadmill, and monitored the blood pressure changes of hypertensive rats monthly before and after exercise until 9 months of exercise training. The results confirmed that starting from 2 months of exercise training, the blood pressure of hypertensive rats was significantly lower than that of sedentary hypertensive rats. During the period of 3 to 9 months of exercise, the blood pressure of rats in the exercise group remained stable and lower than that of the control group (Supplementary Figure S1). Therefore, we adopted a 3-month training time course to explore the correlation between the impact of exercise on hemodynamic parameters of hypertension and vascular remodeling.

**Figure 2:**
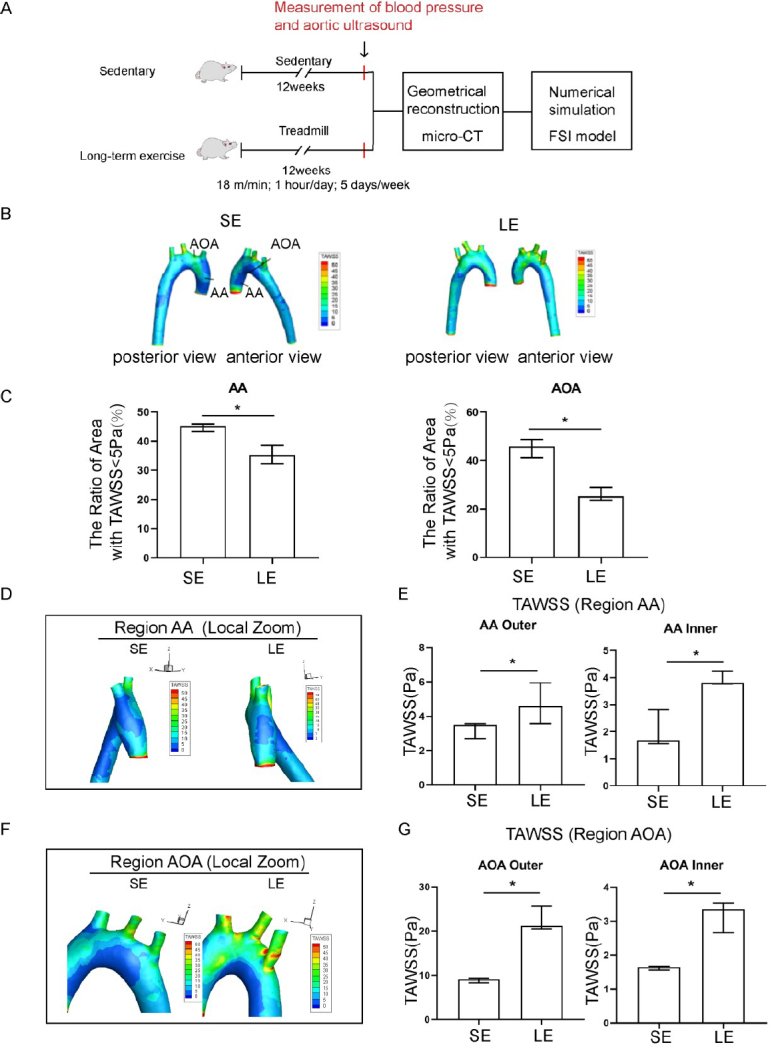
Exercise training induced elevated time-averaged wall shear stress (TAWSS) in the aorta of hypertensive rats. (A) A pattern diagram of the experimental protocol. (B) TAWSS distribution in the aorta of sedentary and long-term exercise trained spontaneously hypertensive rats (SHR). (C) Low TAWSS area (TAWSS < 5 Pa) in the ascending aorta and aortic arch between sedentary and exercise-trained SHR. (D) TAWSS distribution in the ascending aorta of sedentary and exercise-trained SHR with a left view shown. (E) Comparison of the TAWSS of the inner and outer walls of the ascending aorta between sedentary and exercise-trained SHR. (F) TAWSS distribution in the aortic arch of sedentary and exercise-trained SHR, with anterior view. (G) Comparison of the TAWSS of the inner and outer walls of the aortic arch between sedentary and exercise-trained SHR. Data are presented as median (minimum-maximum). *n* = 3. The Mann-Whitney U test was used to assess the difference between the two groups. ^*^P <0.05. AA, ascending aorta; AOA, aortic arch; SE, sedentary; LE, long-term exercise.

In this study, SHR rats were randomly divided into a longterm exercise training group and a sedentary group. Before exercise training, there were no significant differences in weight, heart rate, and blood pressure between the two groups of SHR (Supplementary Table S1). After a 12-week training regime (Figure 2A), the average blood pressure of rats in the longterm exercise training group was significantly lower than that in the sedentary group (Supplementary Table S2, systolic blood pressure, Long-term exercise: 190 (185–193) mmHg *vs*. Sedentary: 204 (203–219) mmHg). The area with the lowest TAWSS (Longterm exercise: 1.15 Pa, Sedentary: 1.25 Pa) was located at the bifurcation of the AOA region and its branches (Figure 2B). The areas with relatively lower TAWSS values (< 5 Pa) were mainly distributed on the inner wall of the AA (AA inner), the inner wall of the AOA (AOA inner), and the branching of the AOA on the outer wall.

Exercise training significantly affected both the absolute value and the spatial distribution of TAWSS as compared to the sedentary group of SHR. High risk regions, defined as the regions with low TAWSS (< 5 Pa), were significantly decreased in samples from the long-term exercise group (Figure 2C). The long-term exercise group also showed a shift in the local spatial distribution of those high-risk regions, as seen from the local magnified images of the AA and AOA region (Figure 2D-2G).

### Evaluating the effect of exercise training on local OSI in the aorta of hypertensive rats using FSI

**Figure 3:**
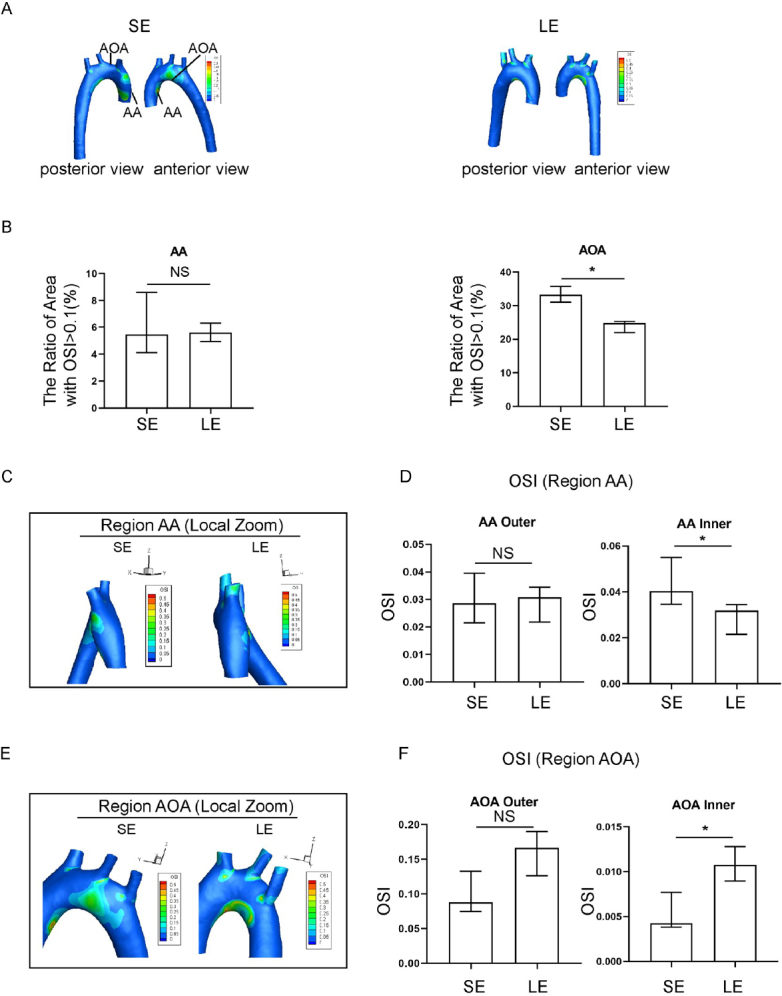
Oscillatory shear index (OSI) in spontaneously hypertensive rats (SHR) in the sedentary and long-term exercise groups. (A) Distribution of aortic OSI in sedentary and long-term exercise groups of SHR rats, with anterior and posterior views. (B) Comparison of high OSI areas (OSI > 0.1) in the ascending aorta (AA) and aortic arch (AOA) between the sedentary and long-term exercise groups of SHR. (C) OSI distribution in the ascending aorta of sedentary and longterm exercise groups of SHR, with a left view shown. (D) Comparison of the OSI on the inner and outer walls of the ascending aorta between the sedentary and long-term exercise groups of SHR. (E) OSI distribution in the aortic arch of sedentary and long-term exercise groups of SHR, with a front view shown. (F) Comparison of the OSI on the inner and outer walls of the aortic arch between the sedentary and long-term exercise groups of SHR. Data are presented as median (minimum-maximum). *n* = 3. The Mann-Whitney U test was used to assess the difference between the two groups. NS, not significant. ^*^P <0.05. AA, ascending aorta; AOA, aortic arch; SE, sedentary; LE, long-term exercise.

Exercise training significantly affected both the absolute value and the spatial distribution of OSI, as compared to the sedentary group. High risk regions, defined as the area of regions with high OSI, were reduced significantly in the long-term exercise group (Figure 3A, 3B). The longterm exercise group also showed a shift in local spatial distribution of the high-risk regions, as seen from the magnified images of the AA and AOA region (Figure 3C-3F). OSI was relatively higher in the areas of high geometric curvature of the AA on the outer side and the AOA inner. On the other hand, the long-term exercise group showed an overall reduced OSI values compared to the sedentary group, especially in the AOA inner region. However, the long-term exercise group had an increase in OSI value in the outer AOA region.

### Evaluating the effect of exercise training on local RRT in the aorta of hypertensive rats using FSI

**Figure 4:**
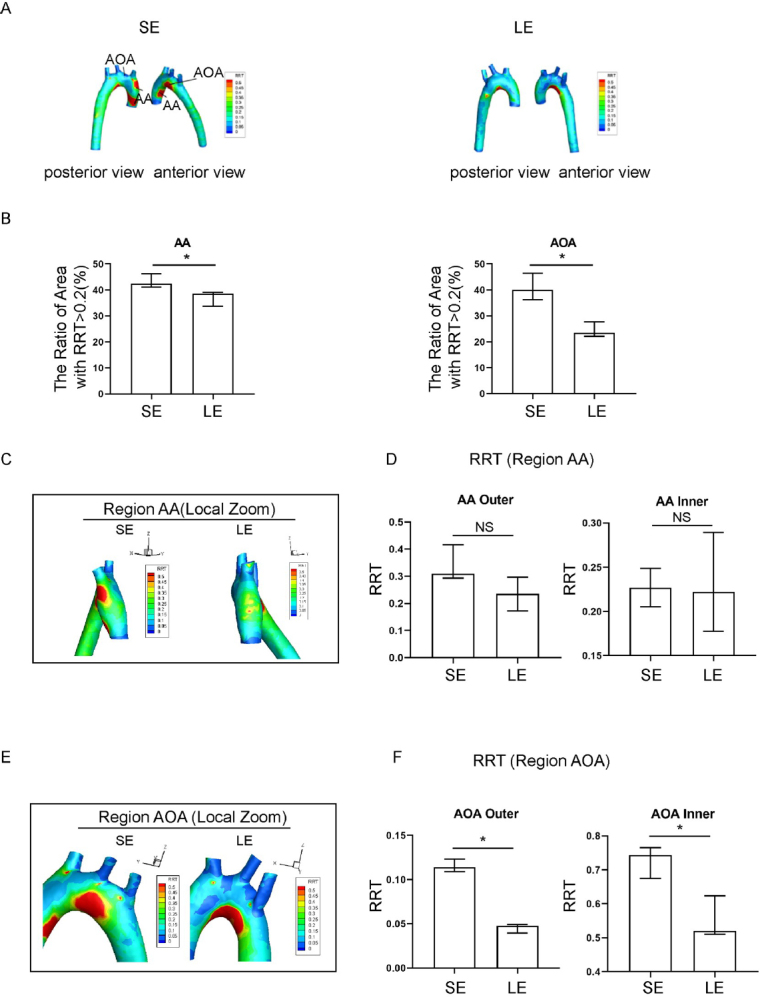
Relative residence time (RRT) in sedentary and long-term exercise spontaneous hypertensive rats (SHR). (A) Distribution of aortic RRT in sedentary and long-term exercise groups of SHR, with anterior and posterior views. (B) Comparison of the areas of high RRT (RRT > 0.2) in the ascending aorta and aortic arch between the sedentary and long-term exercise groups of SHR. (C) RRT distribution in the ascending aorta of sedentary and long-term exercise groups of SHR (left view). (D) Comparison of the mean RRT of the inner and outer walls of the ascending aorta between the sedentary and long-term exercise groups of SHR. (E) RRT distribution in the aortic arch of the sedentary and long-term exercise groups of SHR (front view). (F) Comparison of the mean RRT of the inner and outer walls of the aortic arch between the sedentary and long-term exercise groups of SHR. Data are presented as median (minimum-maximum). *n* = 3. The Mann-Whitney *U* test was used to assess the difference between the two groups. NS, not significant. ^*^P < 0.05. AA, ascending aorta; AOA, aortic arch; SE, sedentary; LE, long-term exercise.

Exercise training significantly affected both the absolute value and spatial distribution of RRT, as compared to the sedentary group. The RRT distribution in the longterm exercise group showed a change in pattern that was reminiscent of the pattern of change for the TAWSS distribution (Figure 4A). The long-term exercise group showed a decrease in RRT value in the outer AA and both inner and outer AOA (Figure 4B), and also a spatial shift of high RRT region towards the AA (Figure 4C-4F). The RRT of the hypertensive rats in the long-term exercise training group was lower than that in the sedentary group at the inner side of the AOA. The RRT of the inner of ascending aorta and aortic arch has a relatively downward trend, which is more consistent with the distribution of TAWSS.

### Exercise training affected the distribution of low wall shear stress and collagen volume fraction in the aortic arch of hypertensive rats

Our previous report confirmed that the level of blood flow shear stress in hypertensive rats is negatively correlated with the local collagen volume fraction of the blood vessels - that is, the local blood vessels with lower shear force in hypertensive rats have higher collagen volume fraction.^[8]^ The above results (Figure 2) suggest that the overall level of TAWSS increases after exercise training. The AA inner region and AOA inner region showed the most significant changes. The AOA outer area shows an increasing trend. To investigate the effect of exercise on collagen volume fraction in the aortic vessels of hypertensive rats, we performed Masson staining on cross-sectional sections of aortic blood vessels and calculated collagen volume fractions for different vascular segments from each treatment group of rats in our experiment. The results showed that long-term exercise training significantly reduced the cross-sectional area, wall thickness, and collagen volume of the aortic wall in hypertensive rats (Supplementary Table S3). In addition, we measured the vascular wall thickness and diameter of the aortic arch using aortic ultrasound to evaluate changes in vascular remodeling, the same results were obtained, long-term exercise training can significantly reduce the thickness of the intima media of the aorta in hypertensive rats (Supplementary Figure S2).

**Figure 5:**
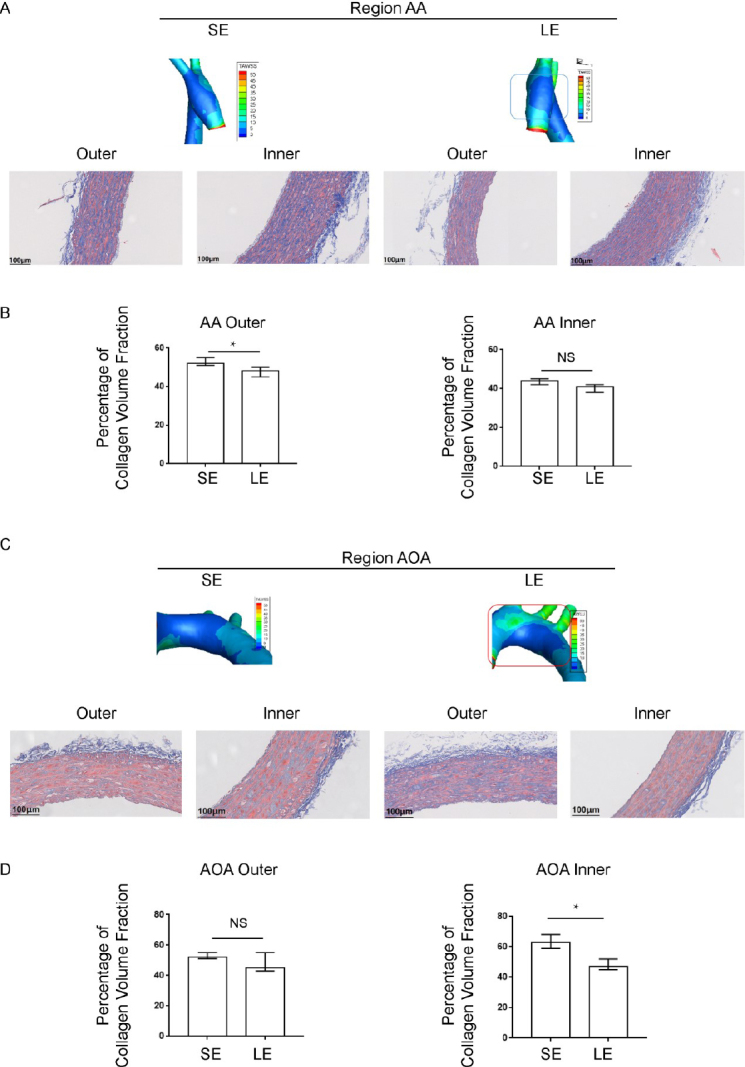
Local distribution of blood flow shear stress and the collagen volume fraction in blood vessels detected by Masson staining. (A) The distribution of wall shear stress on the inner and outer walls of the ascending aorta and (B) the corresponding collagen volume fraction (C) The distribution of wall shear stress on the inner and outer walls of the aortic arch and (D) the corresponding collagen volume fraction. Data are presented as median (minimum-maximum). *n* = 3. The Mann-Whitney U test was used. NS, not significant. ^*^P < 0.05. AA, ascending aorta; AOA, aortic arch; SE, sedentary; LE, long-term exercise.

As shown, we find that long-term exercise training affects collagen deposition in the vascular walls of the AOA in hypertension rats, specifically in the outer AA and inner AOA (Figure 5A-5D). The exercise group showed a significant decrease in collagen volume fraction compared to the sedentary group. In the sedentary group, TAWSS in the outer AA (*r* = -0.646, *P* = 0.023) and inner AOA (*r* = -0.723, *P* = 0.008) was negatively correlated with local collagen volume fraction, while RRT was positively correlated with local collagen volume fraction (*r* = 0.553, *P* = 0.015) (Supplementary Table S4). In the long-term exercise group, the WSS readings in the outer AA (*r* = -0.574, *P* = 0.008) and inner AOA (*r* = -0.397, *P* = 0.032) were negatively correlated with local collagen volume fraction (Supplementary Table S5). Exercise training improves local vascular remodeling and is negatively correlated with the distribution of local WSS, suggesting that exercise training may play a role in reversing vascular remodeling by improving the local hemodynamic environment.

### Exercise training affects the distribution of low wall shear stress regions in the aortic arch of hypertensive rats

**Figure 6:**
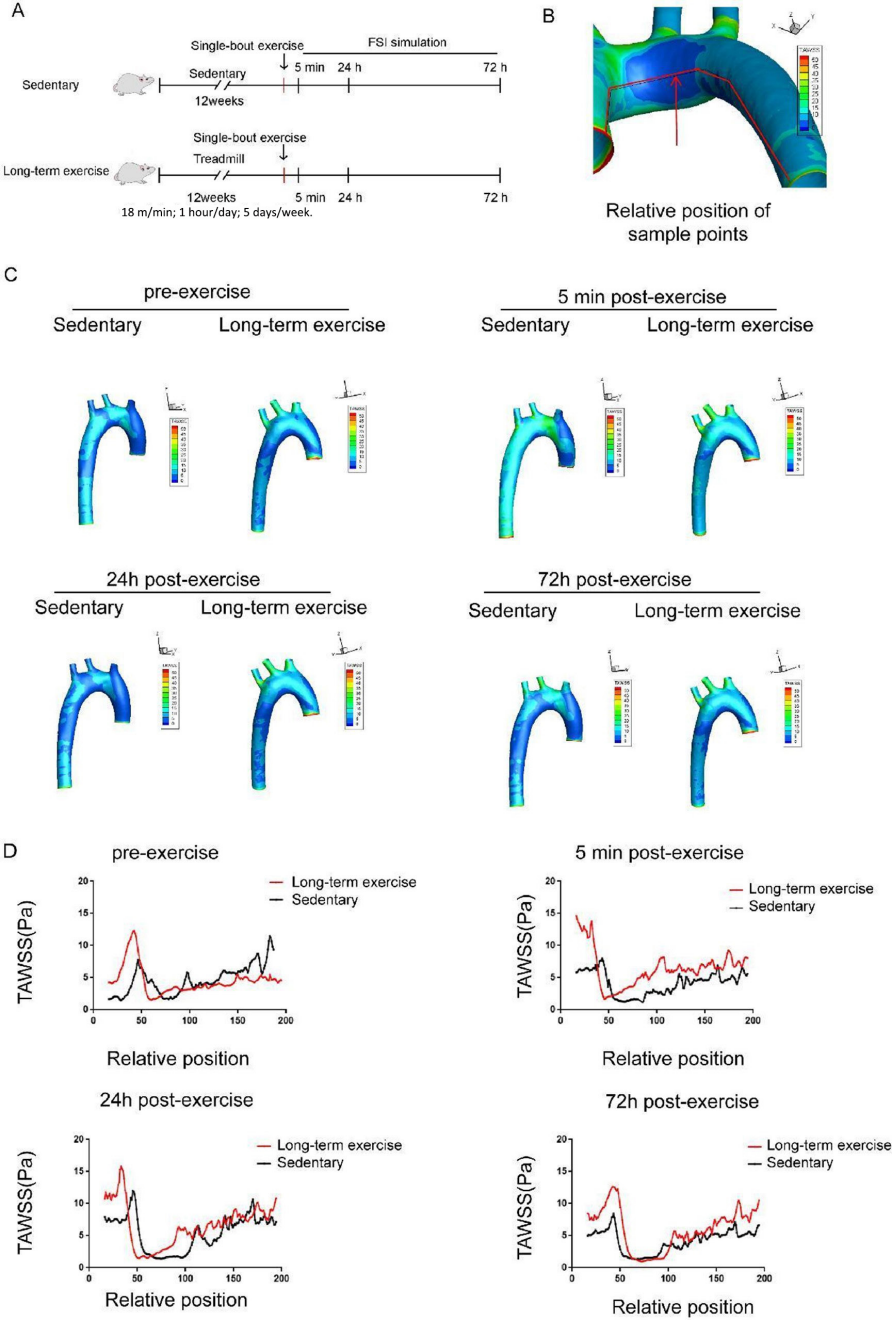
Long-term exercise training affects the distribution of low wall shear stress region in the aortic arch of hypertensive rats. (A) Animal experiment model diagram. (B) Schematic diagram of time-averaged wall shear stress (TAWSS) sampling line along the axis of the aorta. (C) The distribution of TAWSS in the aorta of hypertensive rats before and 5 min, 24 h, and 72 h after a single bout exercise. (D) TAWSS sampling along the medial axis of aorta of the hypertensive rats before and 5 min, 24 h, and 72 h after a single bout exercise. *n* = 3. AA: ascending aorta, AOA: aortic arch.

We speculated that there is a certain maintenance time for the protective hemodynamic effect of exercise training on the aortas of hypertensive rats. Therefore, we conducted a single bout of exercise (18 m/min, running for 1 h) on hypertensive rats in both the sedentary and long-term exercise training groups, and simulated the aortic wall shear force using the FSI method before and at 5 min, 24 h, and 72 h after the single bout of exercise training (Figure 6A). As shown in the line chart, TAWSS parameters corresponding to 200 continuous sampling points were obtained for the inner wall of the aorta (Figure 6B). Due to the influence of the model cutting positions at the inlet and outlet, the first and last 10 sampling points were removed, and a curve was plotted based on the remaining 180 sampling points. As shown in Figure 6C-6D, 5 min or 24 h after training, the peak position shifted towards the AA compared to the sedentary group, with a relative displacement of approximately 3 mm (about 12% relative position).

Five minutes after a single bout exercise, both groups of rats’ aortic TAWSS increased instantaneously. Within 5 min and 24 h after the single bout exercise, there were differences in the area of low TAWSS regions between the two groups of rats; 72 h after the single bout exercise, the low TAWSS areas in the sedentary and long-term exercise groups returned to the same level (Figure 6C-6D). The shift of the low shear stress area (< 5 Pa) shifted towards the AA after exercise (Figure 6D).

## Discussion

In this study, we have demonstrated that data from the FSI simulation method offers a closer approximation of the metrics of actual blood flow, when compared with data from the CFD simulation method, using ultrasound data from rat aortas as a reference. Our study provides the strong evidence that the FSI model, by simulating the interaction between blood flow and the aortic wall, produces a robust physiological simulation result in the AOA region. Without considering the dynamic properties of the aortic wall, CFD simulation tends to overestimate blood flow velocity, and subsequently the WSS, in the AOA. The superiority of FSI over CFD simulation was also validated in the cases of an abdominal aortic aneurysm (AAA) model.^[16]^ Based on these findings, a comprehensive model for FSI provides a more realistic simulated environment by incorporating the elasticity of the aortic wall. Vascular remodeling is actively regulated by not only hemodynamic stimuli, but also by local growth factor signals, vasoactive substances, and other factors.^[17,18]^ In the future, more biological mechanisms could be incorporated into the simulation to develop a more comprehensive physiological simulation model.

Exercise has gained increasing interest by researchers to explore its potential as an effective treatment and preventative action for hypertension, given its advantages as a lower cost intervention that also has fewer potential side effects when compared to the use of antihypertensive drugs.^[19,20]^ There have been numerous studies validating the beneficial effects of exercises through modulating hemodynamic parameters.^[21,22]^ However, exercise prescription is not used extensively due to the uncertainty in its effectiveness and ambiguity about the exact determinants regarding exercise prescription.^[23]^ In the present study, we have, for the first time, showed that exercise not only improved hemodynamic parameter values, but it also resulted in the shift of the spatial distribution of high-risk atherosclerotic areas that featured extreme hemodynamic conditions. Hemodynamic parameters, such as TAWSS, OSI, and RRT, have been calculated to represent the local flow dynamics. Using normotensive rats and SHR as models, we have successfully demonstrated that exercise induced a rise in TAWSS value, reductions in OSI and RRT values, as well as spatial shifts in high-risk areas. This finding offers a mechanistic explanation for how exercise reduces atherosclerotic plaque areas from a hemodynamic force perspective. Particularly, exercise leads to improvement in the absolute values of hemodynamic parameters, and spatial shifts to the area of action of adverse hemodynamic forces.

Exercise training is an important lifestyle modification for improving overall physical health. The guidelines for the prevention and treatment of hypertension from American College of Cardiology/American Heart Association (ACC/ AHA), European Society of Hypertension/European Society of Cardiology (ESH/ESC) indicate that regular exercise is one of the core contents of improving the lifestyle of hypertensive patients.^[24]^ Research has shown that moderate intensity exercise training lasting for 12 weeks has a significant effect on reducing systolic blood pressure and improving vascular remodeling in hypertensive rats.^[25,26]^ We continuously monitored the blood pressure changes of hypertensive rats after long-term moderate intensity exercise training, and found that after 3 months of exercise training, the blood pressure of hypertensive rats can maintain a healthier level, and the vascular remodeling also significantly improved. Based on our research findings, we reconfirm from the perspective of fluid mechanics that, 3-month moderate intensity exercise training can be seen as an alternative or auxiliary means of treating hypertension.

The benefits of exercise have a time effect, and there is still a lack of research data to support the setting of exercise frequency. For healthy adults of all ages, European guidelines recommend conducting at least 5 days and 150 min of moderate intensity exercise training per week.^[24]^ Our study showed that compared to the sedentary group of hypertensive rats, long-term exercise trained hypertensive rats maintain a longer improvement in hemodynamic parameters after a single bout exercise. However, 72 h post exercise, the hemodynamic parameters of the long-term training group returned to a similar level to the sedentary group. The results indicated that a break time of no more than 72 h during exercise training is beneficial for maintaining the improvement effect of exercise on blood flow shear stress. The improvement effect of exercise on hemodynamic parameters in hypertension is related to various factors, including blood circulation efficiency and vascular wall elasticity.^[27]^ In this study, we did not explore the hydrodynamic mechanism of exercise for a longer period of time. In the future, basic and large-scale clinical studies are still needed to explore the time and influencing factors of exercise’s protective effect on hypertension.

The limitations of this study are firstly reflected in the small number of repeated cases in our study. Primarily, given that the research data did not conform to a normal distribution and the sample size was relatively small, non-parametric statistical methods were employed. Additionally, due to current methodological and computational resource constraints, obtaining a large number of cases for fluid-structure coupling calculations was not feasible. In this study, a different approach was taken, involving the analysis of hemodynamic parameters at various time points. Ultimately, to balance computational accuracy and resource efficiency, 3 cases were selected for calculations at each time point. Studies using fluid structure interaction methods to simulate pathological changes often have fewer replicates. Similar studies, such as those investigating shear stress loading on cells during exercise training, have also utilized non-parametric statistical methods.^[28]^ In a study using FSI to simulate aortic dissection, personalized analyses were employed, modeling and analyzing hemodynamic parameters for individual blood vessels.^[29]^ This approach addresses the challenges posed by limited computational resources and offers a nuanced perspective on the dynamics of blood flow in the context of the present research.

The second limitation of this study is that we did not provide a combined intervention of medication and exercise for hypertension. In the treatment of hypertension, medication is the cornerstone, and regular exercise is an effective adjuvant treatment recommended by the guidelines,^[22]^ which is beneficial for preventing and treating hypertension. Our previous research found that after administering medication to SHR to reduce blood pressure and effectively maintain their arterial blood pressure to normal levels, abnormal blood flow shear stress and vascular remodeling still existed in the aorta, indicating that even when hypertension treatment by drugs meets the standard, abnormal blood flow shear stress still exists in the blood vessels.^[8]^ Therefore, on the basis of maintaining normal blood pressure through antihypertensive therapy, intervening in abnormal shear stress and vascular remodeling is an important link in reducing target organ damage in hypertension. This study confirms that long-term exercise training can improve low wall shear stress and vascular remodeling in hypertensive rats, but this rehabilitation treatment requires a long-term chronic onset process. In clinical practice, antihypertensive drugs combined with exercise rehabilitation treatment will further enhance the benefits in hypertension in terms of hemodynamic mechanisms, and this theory needs to be confirmed by future research.

When prescribing exercise, factors such as the duration, intensity, and frequency all present as potential confounds, and the lack of clarity of the therapeutic effects and harms of each of these factors currently complicates its widespread prescription as a treatment for hypertension.^[23,30]^ Our study, based on rat models, has for the first time provided evidence of the dynamic shifts in hemodynamic parameters after exercises of different intervals. Our research results demonstrated that long-term exercise induced continuous improvement in hemodynamic environment post-exercise, which lasted longer compared to a single bout exercise regime. Together, our findings provide a scientific basis for exercise prescription for hypertension. In the future, more experiments with exercises of different duration, intensity, and frequency could be designed to provide further insight into the ideal exercise prescription for hypertensive patients.

## Conclusions

In summary, this study demonstrates the beneficial effect of exercise as a treatment for hypertension from a hemodynamic perspective, using FSI simulation and ultrasound recordings from a rat model of hypertension. We found that exercise not only improved values of hemodynamic parameters, but also shifted the spatial distribution of risk area. In addition, we illustrated the temporal validity of long-term exercise on hemodynamic disorders in hypertension. Taken together, our results further extend the scientific basis for exercise prescription as a viable treatment option for hypertensive patient, and lays the ground work for future studies that define the potential of specific exercise prescriptions.

## Supplementary Material

Supplementary Material
