# Supplementary material for: Spatiotemporal analysis of the effects of exercise on the hemodynamics of the aorta in hypertensive rats using fluid-structure interaction simulation
Source: J Transl Int Med. 2024 Mar 21;12(1):64–77. doi: 10.2478/jtim-2023-0140 (PMC11774211; doi:10.2478/jtim-2023-0140)
Supplement: Supplementary file 1 — Supplementary Material [file jtim-2023-0140_sm.pdf]

Supplementary Materials

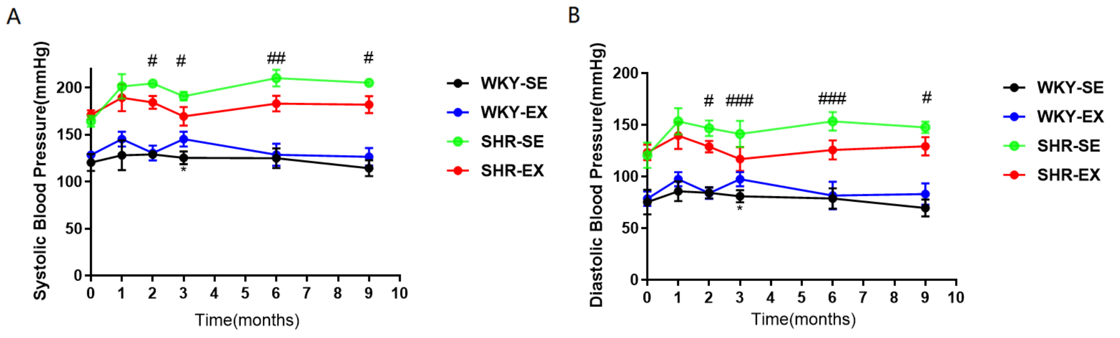

Figure S1. Changes in systolic and diastolic blood pressure in spontaneously hypertensive rats (SHR) and Wistar Kyoto rats (WKY) during the time course of exercise training. The systolic (A) and diastolic blood pressure (B) in the exercise group of hypertensive rats (SHR-EX group) were significantly lower than those in the stationary group of hypertensive rats (SHR-SE group); For non hypertensive control rats (WKYs rats), exercise training has no significant effect on blood pressure. Data are presented as mean ± SD, $n = 5$ in each group. Repeated measures two-way analysis of variance used to compare blood pressure differences at different time points in rats. $^{*}P<0.05$, WKY-EX *vs*. WKY-SE; $^{\#}P < 0.05$, $^{\#\#}P<0.01$, $^{\#\#\#}P<0.001$, SHR-EX *vs*. SHR-SE. SHR-SE, hypertension quiet group; SHR-EX, long-term exercise group with hypertension; WKY-SE, homologous control quiet group; WKY-EX, homologous control long-term exercise group.

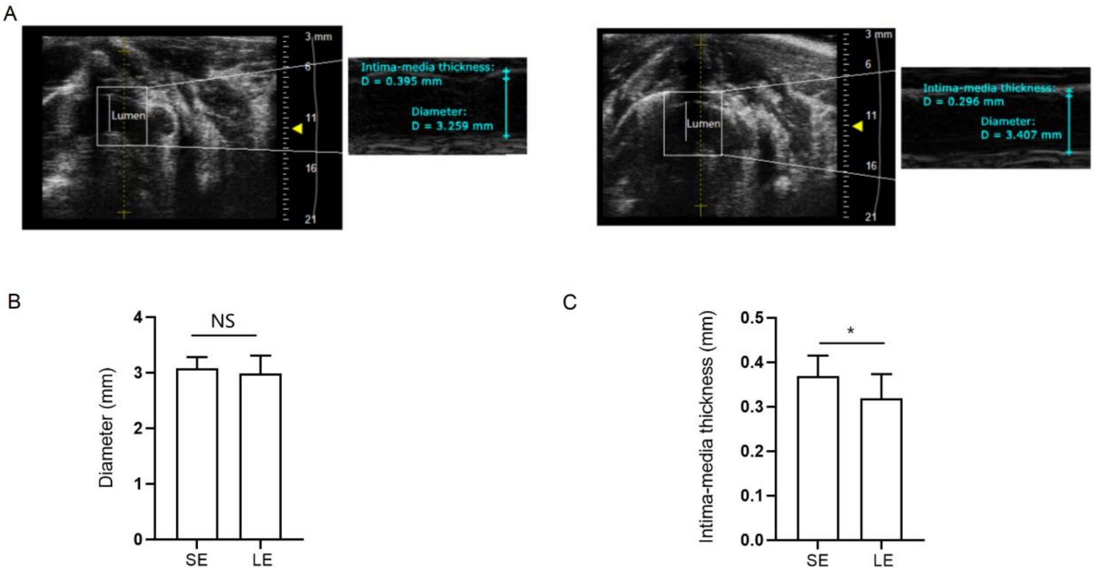

Figure S2. Vascular diameter and intima-media thickness detected by ultrasound imaging in the sedentary and long-term exercise training groups. (A) Ultrasonic measurement of vascular diameter and intima-media thickness in SHRs. (B) Comparison of vascular diameter in the aortic arch (AOA) between the sedentary and long-term exercise groups of SHRs. (C) Comparison of intima-media thickness in the aortic arch (AOA) between the sedentary and long-term exercise groups of SHRs. Data are presented as mean ± SD. n = 12. The t-test was used to assess the difference between the two groups. NS, not significant. *P <0.05. SE, sedentary; LE, long-term exercise.

Table S1. Baseline blood pressure of hypertensive rats before exercise training in the sedentary and long-term exercise training groups

	**Sedentary**	**Long-term exercise**
SBP(mmHg)	168.5±5.1	173.4±8.9
DBP(mmHg)	125.9±9.0	130.1±10.1
HR(bpm)	386.6±24.3	390.5±43.8
BW(g)	183.0±3.7	181.2±3.9

Data are presented as mean ± SD. n = 12. The t-test was used. No significant difference between groups. SBP, systolic blood pressure; DBP, diastolic blood pressure; HR, heart rate; BW, body weight.

Table S2. Blood pressure of hypertensive rats post 3 months of exercise training in the sedentary and long-term exercise training groups

	**Sedentary**	**Long-term exercise**
SBP(mmHg)	204 (203-219)	190 (185-193)*
DBP(mmHg)	163 (150-165)	136 (134-141)*
HR(bpm)	425 (345-453)	325 (313-361)
BW(g)	292 (290-307)	292 (283-295)

Data are presented as median (minimum-maximum) n = 3. The Mann-Whitney U test was used. *P<0.05. SBP, systolic blood pressure; DBP, diastolic blood pressure; HR, heart rate; BW, body weight.

Table S3. Vascular structure of hypertensive rats in the sedentary and long-term exercise training groups

	**Cross-sectional area (mm^2)**	**Diameter (mm)**	**Aorta wall thickness (mm)**	**Collagen volume fraction**
Sedentary	1.664±0.182	1.956±0.178	0.243±0.0315	0.497±0.044
Long-term exercise	1.450±0.161**	1.838±0.139	0.207±0.034*	0.460±0.037*

Data are presented as mean ± SD. n = 12. The t-test was used. *P<0.05, **P<0.01.

Table S4. Correlation analysis between hemodynamic indicators and collagen volume fraction in sedentary hypertensive rats

	**TAWSS**		**OSI**		**RRT**	
	r	***P***	***r***	***P***	***r***	***P***
AA Outer	-0.646	0.023*	0.328	0.298	0.741	0.006**
AA Inner	-0.487	0.108	0.177	0.582	0.263	0.409
AOA Outer	-0.047	0.884	-0.457	0.136	0.696	0.012*
AOA Inner	-0.723	0.008**	0.524	0.080	-0.420	0.175

n = 12. Pearson correlation analysis was used for investigating relationships between hemodynamic indicators and collagen volume fraction, r: Pearson correlation coefficient, *P < 0.05, **P<0.01. AA, ascending aorta; AOA, aortic arch; OSI, oscillatory shear index; RRT, relative residence time; TAWSS, time-averaged wall shear stress.

Table S5. Correlation analysis between hemodynamic indicators and collagen volume fraction in long-term exercise training group hypertensive rats

	**TAWSS**		**OSI**		**RRT**	
	r	***P***	***r***	***P***	***r***	***P***
AA Outer	-0.590	0.043*	-0.109	0.736	0.211	0.510
AA Inner	-0.365	0.244	0.030	0.926	0.112	0.729
AOA Outer	-0.471	0.122	0.372	0.234	-0.215	0.502
AOA Inner	-0.177	0.583	0.254	0.426	-0.143	0.659

$n = 12$. Pearson correlation analysis was used to quantify relationships between hemodynamic indicators and collagen volume fraction, r: Pearson correlation coefficient, $^*P < 0.05$. AA, ascending aorta; AOA, aortic arch; OSI, oscillatory shear index; RRT, relative residence time; TAWSS, time-averaged wall shear stress.
